# Effects of 10-week French contrast training on lower-limb power, short-distance acceleration, and change-of-direction performance in female collegiate basketball players

**DOI:** 10.3389/fphys.2026.1885685

**Published:** 2026-07-08

**Authors:** Limingfei Zhou, Siqi Meng, Zhenxiang Guo, Muxi Zhang

**Affiliations:** 1 School of Strength and Conditioning Training, Beijing Sport University, Beijing, China; 2 Key Laboratory for Performance Training and Recovery of General Administration of Sport of China, Beijing, China; 3 Sports Coaching College, Beijing Sport University, Beijing, China; 4 National Sports Training Center, General Administration of Sport of China, Beijing, China

**Keywords:** acceleration, basketball, change of direction, female athletes, French contrast training, lower-limb power, reactive strength index

## Abstract

**Background:**

Basketball requires repeated jumping, short-distance acceleration, rapid deceleration, and frequent changes of direction, yet it remains unclear whether French contrast training (FCT) produces greater adaptations than equal-load traditional training (ELT) in female collegiate basketball players. This study examined the effects of a 10-week FCT program on lower-limb power, short-distance acceleration, change-of-direction performance, and one-repetition maximum (1RM) back squat performance.

**Methods:**

In this randomized controlled trial, twenty-five female collegiate basketball players were randomly allocated to an FCT group (n = 13) or an ELT group (n = 12). Jump, sprint, change-of-direction, and 1RM back squat outcomes were assessed before and after training and analyzed using two-way repeated-measures ANOVA.

**Results:**

Significant group × time interactions favored FCT for squat jump, countermovement jump, drop-jump height, reactive strength index (all p < 0.001), and eccentric utilization ratio (p = 0.003). Significant group × time interactions were also observed for 10-m sprint time, 10-m maximal velocity, 10-m maximal acceleration, lane agility, and basketball-specific change-of-direction performance (all p < 0.001). *Post hoc* analyses showed significant pre-to-post improvements in the FCT group for all jump-related, sprint, and change-of-direction outcomes (p = 0.035 for eccentric utilization ratio; all other p < 0.001). The ELT group also improved in these outcomes, although the magnitude of improvement was generally smaller. For 1RM back squat, there was a significant main effect of time (p < 0.001), with significant improvements in both groups (both p < 0.001), whereas the group × time interaction was not significant (p = 0.106).

**Conclusions:**

FCT may provide greater benefits than ELT for improving explosive and basketball-specific rapid movement outcomes in female collegiate basketball players. These findings may help strength and conditioning coaches select FCT when the goal is to improve explosive and rapid multidirectional movement performance rather than maximal strength alone.

## 1 Introduction

Basketball is an intermittent team sport characterized by repeated jumping, short-distance acceleration, rapid deceleration, lateral shuffling, and frequent changes of direction (Ibáñez et al., 2024; Kambitta Valappil et al., 2025). These actions occur within short recovery intervals and often under defensive pressure. Match-analysis and field-testing studies indicate that basketball performance depends on the capacity to repeat high-intensity actions, accelerate over short distances, brake effectively, and reorganize body position during multidirectional movement (Barrera-Dominguez et al., 2024). Accordingly, rapid force production and postural control during sport-specific movement are important physical requirements for basketball players (Pleša et al., 2025).

Lower-limb strength and power are central targets in basketball conditioning because they contribute to jumping, braking, sprinting, and reacceleration. Studies in basketball players have reported associations among jump performance, linear sprinting, agility, and change-of-direction (COD) ability (Barrera-Dominguez et al., 2024). Recent evidence also suggests that training methods combining resistance and plyometric stimuli can improve performance variables that are central to basketball, including vertical jump, agility, sprint, and COD outcomes (Wang et al., 2024). These findings are particularly relevant for female collegiate basketball players, who must improve explosive performance while maintaining landing quality and lower-limb alignment (Sánchez-Sixto et al., 2021; Cao et al., 2024).

Resistance training can improve maximal strength and provides an important foundation for force production (Cormier et al., 2020). However, isolated resistance training mainly targets the high-force, low-velocity region of the force-velocity curve and may provide a limited stimulus for rapid force expression (Thapa et al., 2024). By contrast, complex and contrast training methods combine high-load resistance exercise with explosive movements to link force production with rapid movement execution. Systematic reviews and meta-analyses indicate that complex training can improve strength, power, sprint, and change-of-direction outcomes in athletes, including basketball players (Cormier et al., 2020; Lin et al., 2025).

French contrast training (FCT) extends this logic by combining heavy resistance exercise, plyometric exercise, light-load ballistic exercise, and assisted plyometric exercise within the same training sequence (Zhao et al., 2025; Zhou et al., 2026). This approach is intended to expose athletes to multiple force–velocity demands: heavy resistance exercise emphasizes high-force production, plyometric exercise targets rapid eccentric–concentric transition, ballistic exercise emphasizes high-velocity force expression, and assisted plyometric exercise reduces external loading to encourage maximal take-off velocity and short ground-contact actions (Suchomel et al., 2016, Suchomel et al., 2018; Zhao et al., 2025). The sequencing of heavy and explosive exercises is also consistent with the theoretical basis of complex and contrast training, in which a high-load conditioning activity may enhance subsequent explosive performance through post-activation performance enhancement-related mechanisms (Seitz and Haff, 2016; Blazevich and Babault, 2019; Cormier et al., 2020). Several training studies support this rationale. Contrast or complex training has improved jump performance and agility-related outcomes in basketball players (Cao et al., 2024; Wang et al., 2024), and recent FCT studies in other intermittent or explosive sports have reported improvements in vertical jump and sprint performance, with smaller or non-significant advantages for maximal strength (Chen et al., 2025). French contrast training is intended to expose athletes to multiple force–velocity demands within the same sequence: heavy resistance exercise emphasizes high-force production, plyometric exercise targets rapid eccentric–concentric transition, ballistic exercise emphasizes high-velocity force expression, and assisted plyometric exercise reduces external loading to encourage maximal take-off velocity and short ground-contact actions.

Although FCT has been examined in several athletic populations, the current evidence remains heterogeneous. Previous FCT or FCT-related studies have investigated martial arts athletes (Chen et al., 2025), male college badminton players (Zhou et al., 2026), youth soccer players (Thomsen et al., 2026), and female soccer players (Aslan et al., 2026), while broader complex- and contrast-training evidence has also been reported in basketball and other team-sport populations (Latorre Román et al., 2018; Cormier et al., 2020; Wang et al., 2024). Recent FCT-specific reviews and meta-analyses suggest that FCT can improve lower-limb power, sprint performance, and change-of-direction-related outcomes; however, the magnitude and transfer of adaptation appear to vary according to participant characteristics, sport background, training status, intervention duration, and outcome measures (Zhao et al., 2025).

A matched-load comparison is practically important because it helps determine whether the sequencing of heavy resistance, plyometric, ballistic, and assisted plyometric exercises provides benefits beyond completing the same exercises, loads, sets, and repetitions in a traditional blocked structure. This rationale is consistent with evidence from complex and contrast training showing that the sequence and organization of strength–power exercises may influence performance-based adaptations, even when similar training components are used (Cormier et al., 2020; Thapa et al., 2024). This distinction is relevant for strength and conditioning coaches because FCT may require greater technical organization and session planning than traditional equal-load training. Therefore, this study examined the effects of a 10-week FCT program on lower-limb power, short-distance acceleration, change-of-direction performance, and maximal strength in female collegiate basketball players. We hypothesized that FCT would produce greater improvements in jump, sprint, and change-of-direction outcomes than ELT, whereas both programs would improve maximal strength.

## 2 Materials and methods

### 2.1 Participants

An *a priori* sample size estimation was conducted using G*Power software (Heinrich-Heine-Universität Düsseldorf, Düsseldorf, Germany) (Faul et al., 2009). The calculation was based on previous French contrast or complex-training research reporting large effects for countermovement jump performance. Using a repeated-measures ANOVA framework for the group × time interaction, with an assumed effect size of f = 0.40, alpha = 0.05, and power = 0.95, the minimum required sample size was 24 participants. Accordingly, 25 female collegiate basketball players were recruited and successfully completed the study.

After baseline testing, participants were randomly allocated to the FCT group (n = 13) or the ELT group (n = 12) using a computer-generated random number sequence. The randomization sequence was generated after eligibility confirmation and baseline testing. Formal allocation concealment was not implemented because the intervention was conducted within a single university team training environment; this has been acknowledged as a methodological limitation. Baseline characteristics, basketball training experience, and baseline performance outcomes were compared between groups to evaluate group comparability before the intervention. No significant between-group differences were observed in age, height, body mass, or basketball training experience at baseline. All participants were members of a university basketball training program and were familiar with regular basketball practice. Inclusion criteria were: (1) female collegiate basketball player; (2) regular participation in basketball training; (3) ability to perform squat- and jump-based training safely; and (4) no lower-limb injury restricting training or testing during the study period. Participants were excluded if they had a recent lower-limb injury, cardiovascular or neurological condition, or contraindication to high-intensity resistance or plyometric exercise.

The study was conducted in accordance with the Declaration of Helsinki and institutional ethical requirements. All participants were informed of the study purpose, testing procedures, potential risks, and benefits before participation, and written informed consent was obtained before testing and training. The study was approved by the Research Ethics Committee of Beijing Sport University (approval number: 2024433H).

All participants were members of the university varsity basketball team and competed in university-level basketball competitions. During the study period, they maintained their regular basketball training schedule, which consisted of approximately 12 h·week−1 of team-based technical, tactical, and conditioning training.

### 2.2 Study design

A two-group pre-post intervention design was used to compare the effects of 10 weeks of FCT and ELT on lower-limb performance in female collegiate basketball players. Testing was completed before and after the intervention. The main outcomes were grouped as jump-related performance, sprint and change-of-direction performance, and maximal strength. Baseline comparability between groups was examined before the intervention.

Before baseline testing, all participants completed a familiarization session to standardize testing procedures and training techniques (Weakley et al., 2024). Testing sessions were conducted at the same time of day for each participant to reduce potential circadian effects. Participants were instructed to avoid strenuous exercise for 48 h before each testing session and to avoid alcohol and caffeine for 24 h before testing. A standardized warm-up was performed before all performance tests. The same testing order, instructions, equipment, and assessor procedures were used at pre- and post-testing.

### 2.3 Training intervention

Both groups trained for 10 weeks, twice per week, with at least 48 h of recovery between sessions. Each session began with a standardized 10-min warm-up consisting of low-intensity running, dynamic mobility, activation exercises, and movement preparation, and ended with a 5-min cool-down. Training sessions were supervised by strength and conditioning staff to ensure consistent exercise technique, loading, rest intervals, and safety. Before baseline testing, all participants completed one familiarization session 48–72 h before the pre-test. The familiarization session used the same testing order, equipment, verbal instructions, and assessor procedures as the formal testing sessions. Participants performed practice trials for all performance tests, including SJ, CMJ, DJ, 10-m sprint, lane agility, basketball-specific COD, and 1RM back squat procedures. For jump, sprint, and change-of-direction tests, participants completed at least two progressive practice trials followed by one maximal familiarization trial. For the 1RM back squat, participants practiced the standardized squat depth, tempo, and warm-up progression without performing a true maximal attempt. Identical familiarization and testing procedures were used for all participants.

The FCT group performed lower-limb complexes composed of four sequential exercises: a heavy resistance exercise, a plyometric exercise, a light-load ballistic exercise, and an assisted plyometric exercise. This sequence was used to stimulate different regions of the force-velocity curve within the same session. The ELT group performed the same exercises, loads, sets, and repetitions as the FCT group, but in a traditional blocked format in which all sets of one exercise were completed before the next exercise. The detailed 10-week progression, including exercise selection, loading intensity, repetition range, assisted-jump variation, and coaching emphasis, is presented in **Table 1**. Thus, the two interventions were matched for training frequency, exercise content, loading progression, and total volume; the main difference between groups was exercise sequencing.

**Table 1:** Overview of the 10-week French contrast training progression.

Period	Goal	Heavy resistance	Plyometric	Ballistic	Assisted plyometric	Coaching emphasis
Weeks 1-2	Technical adaptation	Back squat, 65%-70% 1RM, 4 reps	Low hurdle jump or CMJ, 3–4 reps	Jump squat, 15%-20% 1RM, 3–4 reps	Assisted CMJ, 3–4 reps	Landing control and stable knee alignment
Weeks 3-6	Progressive loading	Back squat, 70%-80% 1RM, 3–4 reps	Hurdle jump or drop jump, 20–30 cm, 3–5 reps	Jump squat, 20%-25% 1RM, 3–5 reps	Band-assisted jump, 3–5 reps	Increase load only when technique remains stable
Weeks 7-10	Explosive emphasis	Back squat, 80%-85% 1RM, 3 reps	Drop jump or hurdle jump, 30–40 cm, 3–5 reps	Jump squat, 25%-30% 1RM, 3–5 reps	Band-assisted jump, 3–5 reps	Maximal intent without excessive fatigue

Training loads were based on individual 1RM testing and adjusted according to movement quality and session readiness. CMJ, countermovement jump; 1RM, one-repetition maximum.

The FCT progression was designed for female collegiate basketball players, with emphasis on movement quality, landing mechanics, knee alignment, and trunk control. During all jumping exercises, participants were instructed to land softly, avoid excessive knee valgus, maintain trunk control, and complete each repetition with maximal intent. Exercise load, jump height, and assisted-jump tension were adjusted according to technical proficiency and fatigue status. If an athlete showed a technical fault, the load or jump height was reduced and the trial was repeated after adequate rest.

Training attendance was recorded for each participant throughout the intervention. Regular basketball training was maintained during the study period. Both groups followed the same team basketball training schedule, which consisted of approximately 12 h·week−1 of technical, tactical, and conditioning practice. Participants were instructed not to perform additional lower-limb strength or plyometric training outside the prescribed intervention.

### 2.4 Outcome measures

All outcome measures were collected before and after the 10-week intervention. The same assessors, equipment, testing order, and verbal instructions were used at pre- and post-testing. Participants completed a standardized warm-up before testing, consisting of 5 min of low-intensity running, dynamic mobility exercises, and progressive practice trials for the planned tests. For jump, sprint, and change-of-direction tests, participants were instructed to perform each trial with maximal effort. The best valid trial was retained for analysis. A trial was repeated if the participant lost balance, used an incorrect movement pattern, failed to follow the required route, or if a measurement artifact was detected.

#### 2.4.1 Squat jump and countermovement jump

Squat jump (SJ) and countermovement jump (CMJ) were used to assess lower-limb explosive power (Van Hooren and Zolotarjova, 2017). Jump height was measured using a contact platform system (Chronojump Boscosystem, Barcelona, Spain; sampling frequency ≥1000 Hz) (Pueo et al., 2020). Participants stood on the platform with both hands fixed on the iliac crests throughout each trial to minimize the contribution of arm swing. For the SJ, participants began from a static squat position with the knees flexed to approximately 90°, as checked by the assessor. After holding the position briefly to minimize countermovement, participants jumped vertically as high as possible. For the CMJ, participants began from an upright standing position, rapidly descended to a self-selected countermovement depth, and immediately performed a maximal vertical jump. Three maximal trials were completed for each test, with 60 s of passive rest between trials. The best jump height (cm) was used for analysis (Villalón-Gasch et al., 2025).

#### 2.4.2 Drop jump, eccentric utilization ratio, and reactive strength index

Drop-jump (DJ) performance was used to assess reactive strength and stretch-shortening cycle function (Montoro-Bombú et al., 2022). Participants stepped from a 30-cm box onto the contact platform and then jumped vertically as high as possible while minimizing ground contact time. They were instructed to step, rather than jump, from the box; keep both hands on the iliac crests; land with stable lower-limb alignment; and take off immediately after ground contact. Three valid trials were completed, with 60–90 s of passive recovery between trials. DJ height (cm) and ground contact time (s) were recorded. Reactive strength index (RSI) was calculated as DJ height (m) divided by ground contact time (s). The eccentric utilization ratio (EUR) was calculated as CMJ height (cm) divided by SJ height (cm) to describe the contribution of countermovement to vertical jump performance.

#### 2.4.3 10-m sprint test

A 10-m sprint test was used to assess short-distance acceleration. Sprint kinematics were recorded using a pull-cord linear encoder sprint analysis system (Race Analyzer Kit, Chronojump Boscosystem, Barcelona, Spain). Participants started from a split stance 0.5 m behind the starting line to prevent premature triggering. After an auditory cue, participants sprinted maximally through the 10-m distance and were instructed to continue running beyond the finish line to avoid early deceleration. Three maximal trials were completed, with 2–3 min of passive recovery between trials. The best 10-m sprint time (s), maximal velocity (Vmax, m/s), and maximal acceleration (Amax, m/s²) were retained for analysis.

#### 2.4.4 Lane agility test

**Figure 1:**
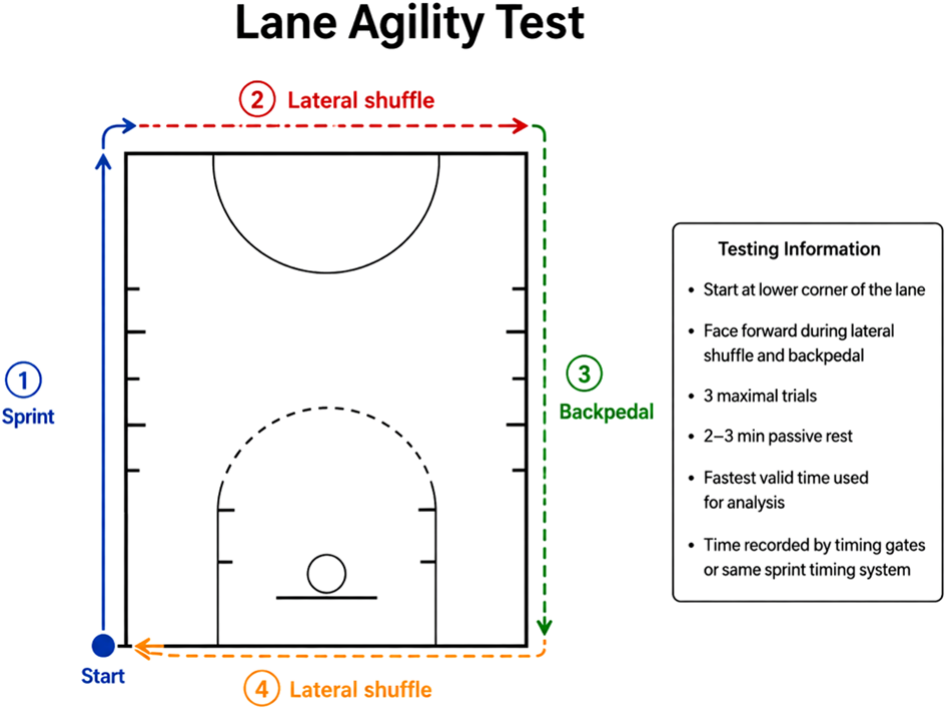
Lane agility test.

The lane agility test was used to assess basketball-related multidirectional movement ability (Sugiyama et al., 2021). The test was performed around the basketball free-throw lane. Participants began at the lower corner of the lane, sprinted forward along the lane line to the free-throw line, shuffled laterally across the free-throw line, backpedaled along the opposite lane line to the baseline, and shuffled laterally across the baseline to the starting side. Participants were required to face forward during the lateral-shuffle and backpedal phases and to complete the course without crossing inside the marked lane boundaries. Completion time was recorded using timing gates or the same timing system used for sprint testing. Three maximal trials were completed, with 2–3 min of passive rest between trials, and the fastest valid time (s) was used for analysis. The layout and movement sequence are shown in **Figure 1**.

#### 2.4.5 Basketball-specific change of direction test

**Figure 2:**
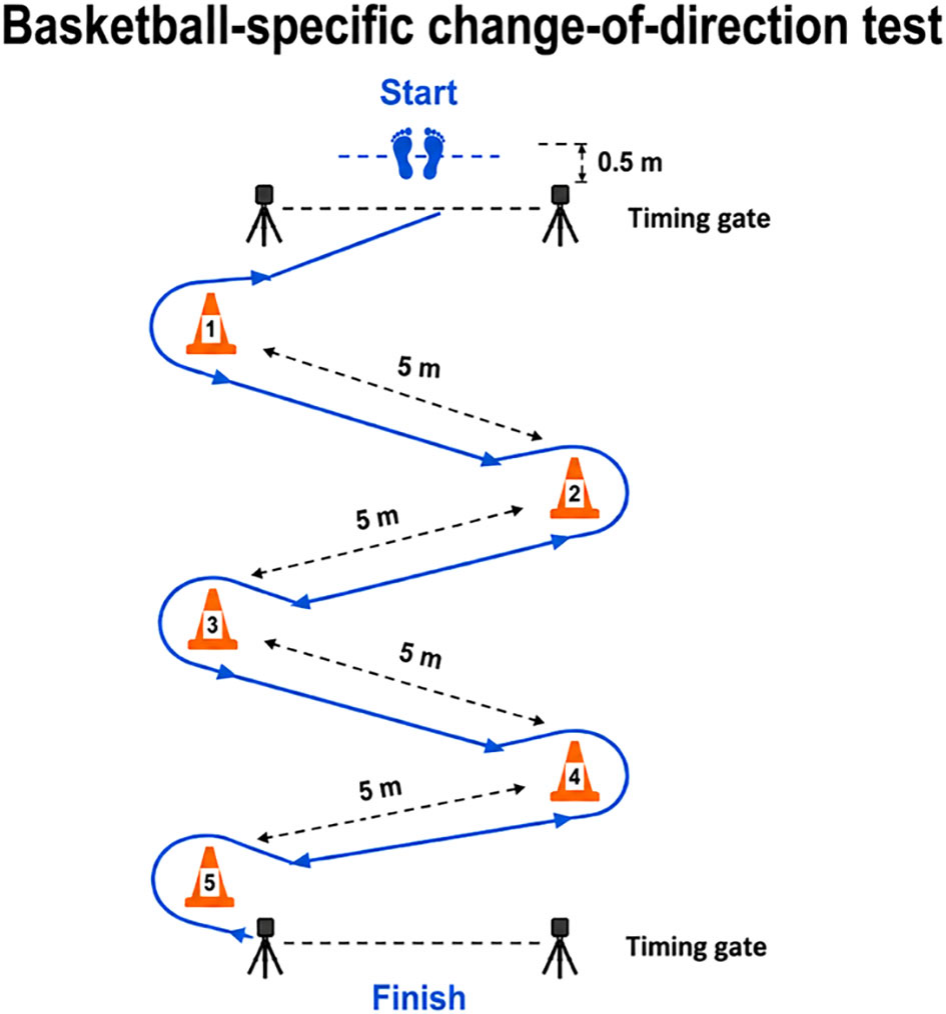
Basketball-specific change of direction test.

A basketball-specific change-of-direction (COD) test was used to assess acceleration, braking, cutting, and reacceleration ability (Wang et al., 2025). The test was organized as a V-cut-style COD course. Five cones were placed 5 m apart in a zig-zag pattern, creating a 25-m course with four directional changes. Participants started 0.5 m behind the first timing gate and sprinted through the course as quickly as possible, cutting around each cone while maintaining the required route. If a participant missed a cone, slipped, or failed to follow the course, the trial was repeated after adequate rest. Three maximal trials were completed with 2–3 min of passive recovery between trials. The fastest valid completion time (s) was retained for analysis. The layout and running route are shown in **Figure 2**.

#### 2.4.6 One-repetition maximum back squat

The one-repetition maximum (1RM) back squat test was used to assess maximal dynamic lower-limb strength (Grgic et al., 2020). Squat depth was standardized so that the thigh reached approximately parallel to the floor, and a depth marker was used to maintain consistent depth across attempts and testing sessions. Participants performed a controlled eccentric phase of approximately 3 s followed by a maximal-effort concentric phase. The warm-up and testing procedure consisted of 10 repetitions with an unloaded or light barbell, 5 repetitions at approximately 50% of the estimated 1RM, 3 repetitions at approximately 75% of the estimated 1RM, and 1 repetition at approximately 90% of the estimated 1RM. The load was then increased by 2.5–10 kg according to participant performance until a failed attempt occurred. The highest load successfully lifted with correct depth and technique within six attempts was recorded as the 1RM. Participants rested for 3–5 min between maximal attempts.

### 2.5 Statistical analysis

All data were analyzed using JASP (version 0.18.3, JASP team, The Netherlands). Data are presented as mean ± standard deviation (SD). Normality of each dependent variable was examined using the Shapiro-Wilk test, and visual inspection was used to identify obvious outliers or data-entry errors. Baseline differences between groups were examined before the intervention using independent-samples tests or non-parametric equivalents when appropriate. Shapiro–Wilk tests and visual inspection did not indicate substantial deviations from normality for the dependent variables. Levene’s tests did not indicate violations of homogeneity of variance; therefore, parametric analyses were retained for all outcomes.

The intervention effects were analyzed using a two-way repeated-measures analysis of variance (ANOVA), with time (pre-test vs. post-test) as the within-subject factor and group (FCT vs. ELT) as the between-subject factor. The primary statistical effect of interest was the group × time interaction, because this term tested whether the magnitude of change differed between FCT and ELT. When a significant main effect or interaction was observed, Bonferroni-adjusted *post hoc* comparisons were used to examine within-group pre-post changes and between-group differences in change. Because the within-subject factor included only two time points, sphericity correction was not required.

Within-group effect sizes were reported as Cohen’s d with 95% confidence intervals (CI). Effect sizes were interpreted as trivial (<0.20), small (0.20-0.49), moderate (0.50-0.79), and large (≥0.80) (Cohen, 2013).

## 3 Results

### 3.1 Baseline characteristics and assumption testing

Baseline characteristics are shown in **Table 2**. No significant differences were observed between the FCT and ELT groups in age, height, body mass, basketball training experience, or any baseline performance outcome (all p > 0.05). Descriptive statistics and ANOVA results for all performance outcomes are summarized in **Table 3**.

**Table 2:** Baseline characteristics of participants.

Variable	FCT group (n = 13)	ELT group (n = 12)	p value
Age (years)	20.47 ± 1.06	20.47 ± 1.34	1.000
Height (cm)	176.08 ± 5.85	173.75 ± 4.30	0.266
Body mass (kg)	66.66 ± 5.72	67.81 ± 4.79	0.590
Basketball training experience (years)	5.17 ± 0.91	5.04 ± 1.24	0.770

Data are presented as mean ± SD. FCT, French contrast training; ELT, equal-load traditional training. p values indicate between-group comparisons at baseline.

**Table 3:** Assessment results for FCT and ELT before and after the 10-week training intervention.

Outcome	FCT Pre	FCT Post	ELT Pre	ELT Post	Time P	Group × time P
SJ (cm)	25.77 ± 2.14	28.55 ± 2.41*#	25.35 ± 2.22	26.44 ± 2.07*	<0.001	<0.001
CMJ (cm)	29.09 ± 2.06	32.75 ± 2.07*#	28.96 ± 2.25	29.78 ± 2.30*	<0.001	<0.001
DJ height (cm)	23.68 ± 1.90	26.26 ± 2.21*#	25.01 ± 2.02	25.69 ± 2.14*	<0.001	<0.001
DJ contact time (s)	0.24 ± 0.02	0.22 ± 0.02*#	0.25 ± 0.01	0.24 ± 0.01*	<0.001	<0.001
EUR	1.13 ± 0.04	1.15 ± 0.06*#	1.14 ± 0.03	1.13 ± 0.03*	0.805	0.003
RSI	0.99 ± 0.10	1.20 ± 0.12*#	1.02 ± 0.11	1.06 ± 0.12*	<0.001	<0.001
10-m sprint (s)	2.05 ± 0.06	1.97 ± 0.07*#	2.02 ± 0.05	1.99 ± 0.05*	<0.001	<0.001
10-m Vmax (m/s)	6.16 ± 0.31	6.43 ± 0.36*#	6.05 ± 0.22	6.15 ± 0.21*	<0.001	<0.001
10-m Amax (m/s^2^)	5.67 ± 0.22	6.04 ± 0.25*#	5.71 ± 0.25	5.82 ± 0.26*	<0.001	<0.001
Lane agility (s)	12.67 ± 0.46	12.26 ± 0.45*#	12.73 ± 0.33	12.60 ± 0.33*	<0.001	<0.001
Basketball COD (s)	6.76 ± 0.13	6.51 ± 0.15*#	6.76 ± 0.16	6.66 ± 0.18*	<0.001	<0.001
1RM back squat (kg)	82.90 ± 8.72	87.67 ± 9.60*	83.55 ± 10.86	87.27 ± 11.43*	<0.001	0.106

Data are presented as mean ± SD. *p < 0.05, significant difference between pre- and post-test within the same group. #p < 0.05, significant difference in change between groups. FCT, French contrast training; ELT, equal-load traditional training; DJ, drop jump; EUR, eccentric utilization ratio; RSI, reactive strength index; Vmax, maximal velocity; Amax, maximal acceleration; COD, change of direction.

### 3.2 Jump-related outcomes

**Figure 3:**
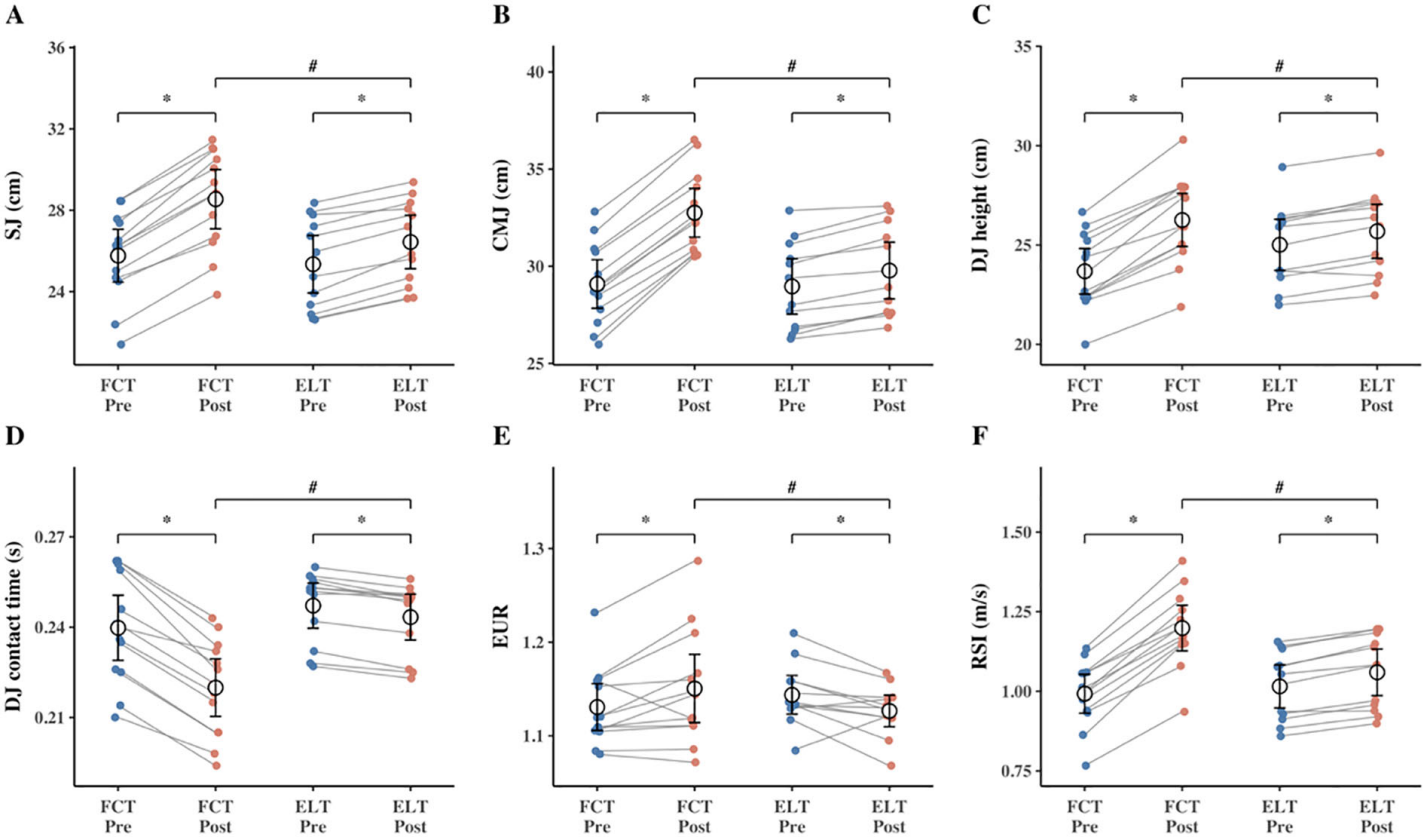
Jump-related outcomes. **(A)** Squat jump (SJ); **(B)** countermovement jump (CMJ); **(C)** drop-jump height; **(D)** drop-jump contact time; **(E)** eccentric utilization ratio (EUR); **(F)** reactive strength index (RSI). *p < 0.05, significant pre-to-post difference within the same group; #p < 0.05, significant between-group difference in change from pre- to post-intervention.

Individual responses and group-level changes in jump-related outcomes are shown in **Figure 3**. For jump-related outcomes, two-way repeated-measures ANOVA showed significant time effects and significant group × time interactions for SJ, CMJ, DJ height, and RSI (all p < 0.001). For EUR, the main effect of time was not significant (p = 0.805), whereas the group × time interaction was significant (p = 0.003). *Post hoc* analyses showed that, in the FCT group, SJ (p < 0.001, d = 4.818, 95% CI [2.834, 6.789]), CMJ (p < 0.001, d = 7.351, 95% CI [4.397, 10.297]), DJ height (p < 0.001, d = 3.292, 95% CI [1.874, 4.691]), EUR (p = 0.035, d = 0.660, 95% CI [0.046, 1.252]), and RSI (p < 0.001, d = 4.346, 95% CI [2.539, 6.137]) changed significantly from baseline to post-intervention. In the ELT group, significant changes were also observed in SJ (p < 0.001, d = 2.866, 95% CI [1.546, 4.163]), CMJ (p < 0.001, d = 2.180, 95% CI [1.105, 3.228]), DJ height (p < 0.001, d = 1.940, 95% CI [0.946, 2.906]), EUR (p = 0.033, d = 0.702, 95% CI [0.054, 1.325]), and RSI (p < 0.001, d = 2.430, 95% CI [1.267, 3.567]). EUR increased slightly in the FCT group but decreased slightly in the ELT group, contributing to the significant interaction. Overall, improvements in SJ, CMJ, DJ height, and RSI were greater in the FCT group than in the ELT group.

### 3.3 Sprint and change-of-direction outcomes

**Figure 4:**
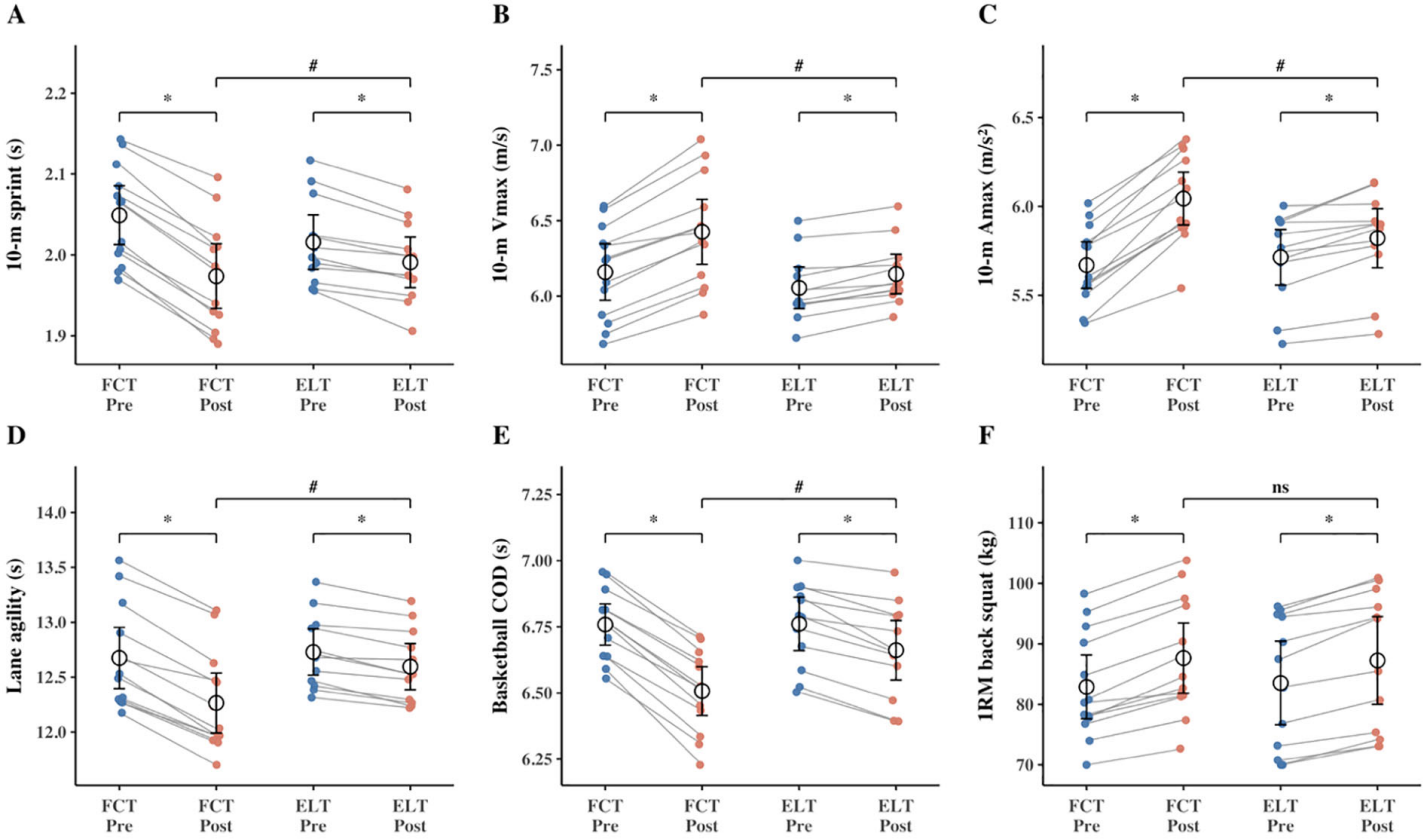
Sprint, COD, and strength outcomes. **(A)** 10-m sprint time; **(B)** 10-m maximal velocity (Vmax); **(C)** 10-m maximal acceleration (Amax); **(D)** lane agility; **(E)** basketball-specific change of direction (COD); **(F)** one-repetition maximum (1RM) back squat. *p < 0.05, significant pre-to-post difference within the same group; #p < 0.05, significant between-group difference in change from pre- to post-intervention; ns, non-significant between-group difference in change.

Individual responses and group-level changes in sprint, change-of-direction, and maximal strength outcomes are shown in **Figure 4**. For sprint and change-of-direction outcomes, two-way repeated-measures ANOVA showed significant time effects and significant group × time interactions for 10-m sprint time, 10-m Vmax, 10-m Amax, lane agility, and basketball-specific COD (all p < 0.001). In the FCT group, significant improvements were observed in 10-m sprint time (p < 0.001, d = 5.116, 95% CI [3.019, 7.201]), 10-m Vmax (p < 0.001, d = 2.989, 95% CI [1.679, 4.278]), 10-m Amax (p < 0.001, d = 3.477, 95% CI [1.992, 4.944]), lane agility (p < 0.001, d = 3.998, 95% CI [2.321, 5.659]), and basketball-specific COD (p < 0.001, d = 4.641, 95% CI [2.724, 6.544]) from baseline to post-intervention. In the ELT group, significant improvements were also observed in 10-m sprint time (p < 0.001, d = 1.867, 95% CI [0.897, 2.808]), 10-m Vmax (p < 0.001, d = 2.237, 95% CI [1.142, 3.305]), 10-m Amax (p < 0.001, d = 1.438, 95% CI [0.603, 2.242]), lane agility (p < 0.001, d = 2.065, 95% CI [1.029, 3.073]), and basketball-specific COD (p < 0.001, d = 2.131, 95% CI [1.072, 3.162]) from baseline to post-intervention. The FCT group showed greater improvements than the ELT group across all sprint and change-of-direction variables.

### 3.4 Maximal strength

For maximal strength, two-way repeated-measures ANOVA showed a significant main effect of time on 1RM back squat (p < 0.001), whereas the group × time interaction was not significant (p = 0.106). Within-group analyses showed that 1RM back squat increased significantly in both the FCT group (p < 0.001, d = 2.845, 95% CI [1.586, 4.082]) and the ELT group (p < 0.001, d = 2.593, 95% CI [1.372, 3.790]) from baseline to post-intervention. Thus, both programs improved maximal strength, but the between-group difference in strength gain was not statistically significant.

## 4 Discussion

The main findings showed that 10 weeks of FCT produced larger improvements than ELT in jump-related, sprint, and change-of-direction outcomes in female collegiate basketball players. This pattern was observed for SJ, CMJ, drop-jump height, drop-jump contact time, RSI, 10-m sprint variables, lane agility, and basketball-specific COD performance. In contrast, 1RM back squat increased in both groups, but the group × time interaction was not significant. These findings are consistent with the broader complex- and contrast-training literature, which shows that combined resistance and plyometric methods are particularly effective for improving explosive and rapid movement outcomes in team-sport athletes and basketball players (Cormier et al., 2020; Wang et al., 2024).

The improvements in jump-related outcomes are consistent with previous basketball training studies. Contrast training has been reported to improve vertical jump, sprinting, and agility performance in young basketball players (Latorre Román et al., 2018), and plyometric or combined plyometric training has improved countermovement jump performance and jump biomechanics in female basketball players (Sánchez-Sixto et al., 2021). Modified complex training has also improved neuromuscular performance in basketball players (Freitas et al., 2019), while earlier complex-training work supports its use for improving explosive strength in young basketball players (Santos and Janeira, 2008). The present findings extend this evidence by showing that a French contrast sequence produced greater improvements in SJ, CMJ, DJ height, DJ contact time, and RSI than an equal-load blocked training format.

One explanation is that the FCT sequence provided a broader spectrum of force–velocity training stimuli than ELT. These adaptations may be partly explained by the interaction between force–velocity spectrum exposure and post-activation performance enhancement-related mechanisms. In the FCT intervention, the heavy resistance exercise may acutely increase neuromuscular activation and motor-unit recruitment, thereby preparing subsequent plyometric and ballistic tasks to be performed with greater movement intent (Seitz and Haff, 2016; Blazevich and Babault, 2019; Cormier et al., 2020). Over repeated sessions, this sequencing may help athletes develop the ability to express force rapidly across braking, propulsion, and short ground-contact actions. Basketball jumping actions require rapid force production and an efficient transition from eccentric braking to concentric propulsion. The heavy resistance component may increase force-production capacity, whereas plyometric, ballistic, and assisted jumping tasks emphasize take-off velocity, short ground-contact time, rate of force development, and stretch-shortening cycle function (Suchomel et al., 2016, Suchomel et al., 2018; Ramirez-Campillo et al., 2023; Zhao et al., 2025). The larger improvement in RSI suggests that FCT may have enhanced reactive strength and stretch-shortening cycle function, allowing athletes to transition more efficiently from eccentric braking to concentric propulsion under short ground-contact conditions. This adaptation is particularly relevant to basketball-specific actions such as rebounding, close-out defense, rapid defensive transitions, and repeated change-of-direction movements (Ramirez-Campillo et al., 2023).

The sprint and change-of-direction findings are also consistent with previous research. Short-distance acceleration depends on rapid horizontal force production, whereas COD performance requires braking, reorientation, and reacceleration. In female basketball athletes, strength characteristics contribute to COD and agility performance (Spiteri et al., 2014). More broadly, systematic review evidence indicates that several training forms, including strength and plyometric training, can improve COD ability in court- and field-based sports (Falch et al., 2019). Basketball-specific work in adolescent female players also suggests that plyometric training in different movement planes can improve change-of-direction speed and power (McCormick et al., 2016). The larger improvements in 10-m sprint time, Vmax, Amax, lane agility, and basketball-specific COD support the practical value of FCT for rapid movement tasks frequently required in basketball.

The maximal-strength result provides an important boundary for interpreting the study. Both training programs improved 1RM back squat, but FCT did not produce a significantly greater increase than ELT. This pattern is consistent with recent evidence suggesting that FCT may be more effective for explosive lower-limb outcomes than for producing clear additional gains in maximal strength when compared with other structured strength-power approaches (Zhao et al., 2025). Recent FCT studies in youth soccer and female soccer also indicate that FCT or FCM can improve sprint-, agility-, or jump-related outcomes, but the transfer to all speed or strength outcomes is not always uniform (Aslan et al., 2026; Thomsen et al., 2026). It also agrees with the logic of the present design, because both groups completed comparable heavy resistance loading. Therefore, maximal strength adaptation probably reflected the resistance-loading component shared by both programs, whereas the additional advantage of FCT appeared in outcomes requiring rapid force expression.

Findings from modern pentathlon studies further support this interpretation. Complex training following or compared with resistance training has improved strength and power outcomes in elite modern pentathletes, but these studies also emphasize that applied training designs should be interpreted cautiously because adaptations may reflect accumulated neuromuscular preparation rather than the isolated effect of one training component (Qiao et al., 2022; Liu et al., 2026). In the present study, the matched-load comparison helps reduce this concern. However, because direct neuromuscular or biomechanical measures were not collected, the proposed mechanisms remain theory-driven interpretations rather than confirmed physiological explanations.

From a practical perspective, coaches can consider FCT when the training goal is to improve jumping, acceleration, and change-of-direction performance in female collegiate basketball players. The method should be implemented progressively, with attention to landing mechanics, knee alignment, trunk control, and recovery. It is most appropriate when athletes have sufficient technical competency in squatting and jumping tasks. When the main goal is maximal strength development, however, equal-load traditional resistance training may provide similar benefits over the same training period.

Several limitations should be considered. First, the sample size was relatively small, which limits statistical power and generalizability. Second, the study did not include a non-training control group; therefore, the observed improvements cannot be separated completely from the potential effects of regular basketball training, repeated testing, or normal seasonal development. Third, outcome assessors were not blinded to group allocation, although standardized testing procedures, fixed testing order, identical equipment, and predefined validity criteria were used to reduce potential assessment bias. Fourth, although a familiarization session was conducted, repeated exposure to the performance tests may have contributed to learning effects. Fifth, study-specific minimal detectable change values were not calculated because repeated baseline reliability testing was not conducted; therefore, the extent to which each individual change exceeded measurement error cannot be determined definitively. Sixth, direct neuromuscular, biomechanical, or physiological measurements were not collected, limiting interpretation of the mechanisms underlying the observed performance changes. Seventh, the study focused on female collegiate basketball players, so the findings should not be transferred directly to male athletes, youth athletes, or professional players. Finally, the 10-week intervention and absence of long-term follow-up limit conclusions regarding longer-term adaptation and retention of training effects. Future studies should include larger samples, non-training control groups, longer intervention periods, monitoring of background basketball training load, and direct biomechanical or neuromuscular measurements.

## 5 Conclusion

Under the present training conditions, a 10-week FCT program may provide greater benefits than equal-load traditional training for improving lower-limb power, short-distance acceleration, and change-of-direction performance in female collegiate basketball players. Both FCT and ELT improved maximal strength, but FCT did not produce a significantly greater increase in 1RM back squat. These findings suggest that, when resistance loading is matched, FCT may be better suited to improving explosive and basketball-specific rapid movement performance than to producing additional maximal-strength gains.

## Data Availability

The original contributions presented in the study are included in the article/supplementary material. Further inquiries can be directed to the corresponding authors.
